# Trivalent Disulfide Unit-Masked System Efficiently Delivers Large Oligonucleotide

**DOI:** 10.3390/molecules29174223

**Published:** 2024-09-05

**Authors:** Lei Wang, Xiao Liu, Yiliang Wu, Zhaoyan Ye, Yiru Wang, Shengshu Gao, Hao Gong, Yong Ling

**Affiliations:** School of Pharmacy, Jiangsu Province Key Laboratory for Inflammation and Molecular Drug Target, Nantong University, Nantong 226001, China; lx199803@126.com (X.L.); 13815218649@163.com (Y.W.); jackcooper1128@163.com (Z.Y.); yrjw779@126.com (Y.W.); 19551223085@163.com (S.G.); 18915458067@163.com (H.G.)

**Keywords:** oligonucleotide delivery, disulfide unit, cellular uptake

## Abstract

Oligonucleotide drugs are shining in clinical therapeutics, but efficient and safe delivery systems severely limit their widespread use. A disulfide unit technology platform based on dynamic thiol exchange chemistry at the cell membrane has the potential for drug delivery. However, the alteration of the disulfide unit CSSC dihedral angle induced by different substituents directly affects the effectiveness of this technology and its stability. Previously, we constructed a trivalent low dihedral angle disulfide unit that can effectively promote the cellular uptake of small molecules. Here, we constructed a novel disulfide unit-masked oligonucleotide hybrid based on a low dihedral angle disulfide unit, motivated by prodrug design. Cellular imaging results showed that such a system exhibited superior cellular delivery efficiency than the commercial Lipo2000 without cytotoxicity. The thiol reagents significantly reduced its cellular uptake (57–74%), which proved to be endocytosis-independent. In addition, in vivo distribution experiments in mice showed that such systems can be rapidly distributed in liver tissues with a duration of action of more than 24 h, representing a potential means of silencing genes involved in the pathogenesis of liver-like diseases. In conclusion, this trivalent disulfide unit-masked system we constructed can effectively deliver large oligonucleotide drugs.

## 1. Introduction

Oligonucleotide (ODN) therapy, as a new technology of biological therapy, provides an excellent ‘arsenal’ for the current evolving human disease spectrum and provides a theoretical basis and technical guarantee for upgrading the level of clinical treatment [1,2,3,4,5,6]. However, delivering these bulky ODN drugs into target cells is still a challenging task [7,8]. Therefore, it has also inspired researchers to develop novel and efficient means of delivery [9,10,11], such as the most widely used liposome nanoparticles [12,13]. Typically, positively charged liposomes interact with negatively charged ODN drugs to form nanocomplexes, which then enter the cell through the process of endocytosis. However, some defects have been exposed to seriously limit their application in clinical therapy [14,15], such as the following: low endosomal escape efficiency and reduced silencing effect on target genes; high cytotoxicity due to their local enrichment of positive charge; and high molecular weight, making the formation of homogeneous nanocomplexes difficult.

Recently, the development of a drug delivery system based on dynamic thiol exchange chemistry at the cell membrane has been widely used [16,17,18]. Compared with liposomal nanoparticles, this system has a small molecular weight of disulfide units, a simple control, and less charge-free toxicity. However, the structural differences of disulfide units seriously affect the delivery effect [19]. For example, disulfide units with high CSSC dihedral angles accelerate the exchange rate with thiol groups on the cell membrane to facilitate delivery, but are accompanied by reduced structural stability. Previously, we have successfully improved the delivery efficiency of low dihedral angle disulfide units for small molecules by more than 100-fold through trivalent coupling [20], but the effect of this chemical modification on the delivery of large ODN drugs is still not known.

**Figure 1:**
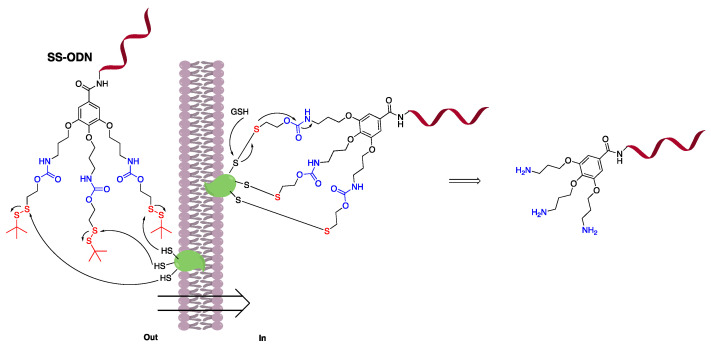
Brief description of disulfide unit-masked oligonucleotide (SS-ODN) strategy used. The red marks represent disulfide unit reaction parts. The blue marks represent prodrug release parts.

Disulfide unit delivery systems enter the cell to release drugs with naked thiol groups in the presence of glutathione. To avoid potential cellular irritation by the naked thiol group, we used a prodrug-like design strategy that closes the thiol group and releases a relatively mild amino substituent (Figure 1) under mild conditions compared to other ways [21]. Therefore, in order to further explore the wide application of trivalent low dihedral angle disulfide units in large ODN drug delivery, we constructed disulfide unit-masked and -coupled ODN sequence (SS-ODN) using tert-butyl as an example, and verified the delivery effect by cell and animal experiments.

## 2. Results and Discussion

### 2.1. Chemistry

#### 2.1.1. Synthesis of Disulfide Unit-Masked Phosphoramidite Monomer

**Scheme 1:**
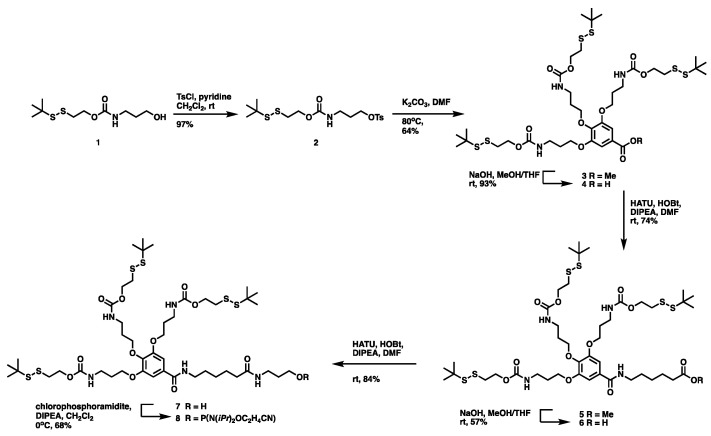
Synthesis of disulfide unit-masked phosphoramidite monomer.

The synthetic scheme for disulfide unit-masked phosphoramidite monomer is illustrated in Scheme 1. Firstly, the disulfide unit-masked alcohol **1** was prepared according to the reported literature [22]. The tosyl chloride reagent was then used to activate the hydroxyl group at the terminal to obtain an intermediate **2**, which then reacted with methyl trihydroxybenzoate to give the key intermediate **3** with moderate yield. After the hydrolysis of methyl benzoate under NaOH solution, the linkers, such as 6-aminohexanoate and 3-amino-1-propanol, were extended by a classical amide condensation reaction using HATU to yield the key alcohol intermediate **7**, which was then transferred to the final disulfide unit-masked phosphoramidite monomer **8** by the conventional phosphoramidite method. The ^1^H NMR, ^13^C NMR, and HRMS spectra of all the compounds are provided in Figures S1–S5.

#### 2.1.2. Synthesis of Disulfide Unit-Masked and -Conjugated Oligonucleotide

**Scheme 2:**
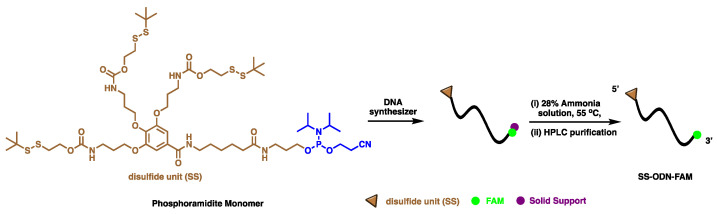
Synthesis of disulfide unit-conjugated oligonucleotide (SS-ODN-FAM).

The synthetic scheme for disulfide unit-masked and -conjugated oligonucleotide is illustrated in Scheme 2. Firstly, the final disulfide unit-masked phosphoramidite monomer **8** was used to synthesize the disulfide unit-conjugated oligonucleotide with FAM label at 3′-terminal (SS-ODN-FAM) on an automated DNA synthesizer (Nihon Techno Service Co., Ltd., Ibaraki, Japan) using standard phosphoramidite chemistry. High-purity SS-ODN-FAM was obtained by HPLC purification (Figure S6) and the structural integrity of the SS-ODN-FAM was confirmed by MS measurements (Figure S7). As the control, non-disulfide unit-conjugated oligonucleotide (ODN-FAM) was purchased from Suzhou Biosyntech Co., Ltd (Suzhou, China). Finally, the UV absorption value of FAM at 520 nm in sodium tetraborate buffer (pH = 9.18) was used to calculate the exact concentration of the SS-ODN-FAM and ODN-FAM.

### 2.2. Biological Activity

#### 2.2.1. Cellular Uptake Evaluation of SS-ODN-FAM Probe Compared with Lipo2000

**Figure 2:**
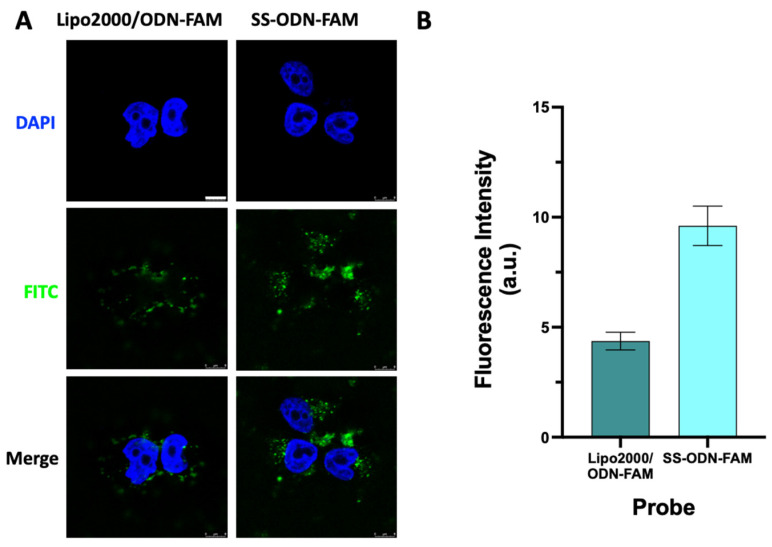
(**A**) CLSM images of HeLa S3 cells after incubation with 1 μM SS-ODN-FAM probe and Lipo2000/ODN-FAM (1 μM) complex for 4 h. Nuclei were stained with DAPI. (**B**) Quantitative analysis of fluorescence intensity. Scar bar: 8 µm. The blue marks represent DAPI staining. The green marks represent the fluorescent signal of FAM.

A number of cell experiments were conducted to assess the fluorescent probe’s biological activity and toxicity. Firstly, the disulfide-mediated cellular delivery capacity of the SS-ODN-FAM probe was evaluated by cellular uptake assay and compared with the commercial oligonucleotide transfection reagent Lipo2000. Taking HeLa S3 cells as an example, the SS-ODN-FAM probe (1 μM, in DMEM) was incubated with HeLa S3 cells alone at 37 °C for 4 h. In addition, the commercial Lipo2000 was incubated with ODN-FAM (1 μM) for 20 min in vitro to form a stable Lipo2000/ODN-FAM complex and incubated with HeLa S3 cells under the same conditions. The uptake capacity of the HeLa S3 cells for different fluorescent probes was monitored by a confocal laser scanning microscope (CLSM). From the imaging results, when ODN-FAM was transfected with the Lipo2000, significant fluorescence excitation was found in the cytoplasm, and no significant fluorescence signals were seen in the nucleus (Figure 2A), similar to the effects of the use of Lipo2000 that are now commonly reported. When the disulfide unit-masked SS-ODN-FAM probe was used, similarly, significant fluorescence excitation was observed in the cytoplasm (Figure 2A). The above results indicate that ODN can be delivered to the cell interior even without the assistance of transfection reagents, relying only on disulfide-mediated covalent exchange chemistry at the cell membrane. As shown by the preliminary analysis of fluorescence intensity (Figure 2B), the disulfide unit-mediated cellular uptake efficiency was approximately 2.2 times higher than that of the commercial reagent Lipo2000. In addition, in order to more clearly compare the scope of the application of this SS-ODN-FAM probe, it was incubated with four other different cell lines (MDA-MB-231, HepG2, HT29, and MCF-7) and explored for its delivery effect. It was clear that both the Lipo2000 and SS-ODN-FAM probes were able to enter the cells efficiently (Figures S8A,B and S9A,B) with strong cellular applicability. Similarly, preliminary analysis of the fluorescence intensity showed that the disulfide unit-masked SS-ODN-FAM exhibited better cellular delivery compared with the Lipo2000, which was approximately 1.1–1.7-fold more efficient (Figures S8C and S9C). Considering the differences in the expression levels of thiol-containing proteins on the cell membrane or the redox state of thiol groups [23,24,25,26], disulfide unit-masked SS-ODN-FAM showed slight differences in different tumor cells. For example, the fluorescence intensity was significantly higher in HepG2 (Figure S8B) as well as HT29 cells (Figure S9A). Furthermore, Lipo2000 can deliver ODN into the nucleus in individual cell systems (such as HepG2, HT29, and MCF-7, Figures S8 and S9), with the potential to deliver oligonucleotide drugs with sites of action in the cytoplasm or nucleus. Unfortunately, the disulfide unit-masked SS-ODN-FAM probe was heavily enriched in the cytoplasm, with trace fluorescence excitation found only in the nuclei of HT29 and MCF-7 cells (Figure S9A,B). Therefore, this disulfide unit-masked SS-ODN-FAM system may have good potential in delivering oligonucleotide drugs silencing cytoplasmic targets.

**Figure 3:**
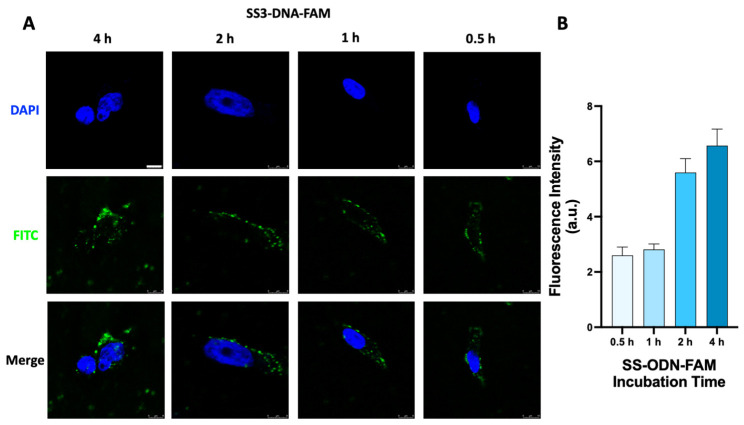
(**A**) CLSM images of HeLa S3 cells after different incubation times with 1 μM SS-ODN-FAM probe. Nuclei were stained with DAPI. (**B**) Quantitative analysis of fluorescence intensity. Scar bar: 8 µm. The blue marks represent DAPI staining. The green marks represent the fluorescent signal of FAM.

**Figure 4:**
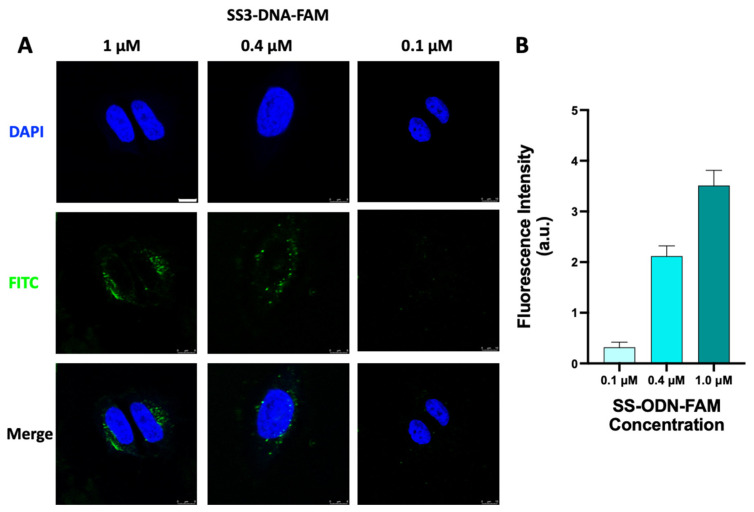
(**A**) CLSM images of HeLa S3 cells after 1 h incubation with different concentrations of SS-ODN-FAM probe. Nuclei were stained with DAPI. (**B**) Quantitative analysis of fluorescence intensity. Scar bar: 8 µm. The blue marks represent DAPI staining. The green marks represent the fluorescent signal of FAM.

Next, the cellular uptake process of the SS-ODN-FAM probe in HeLa S3 cells was investigated for different periods. The SS-ODN-FAM probe (1 μM, in DMEM) was incubated with HeLa S3 cells at 37 °C for different times (0.5, 1, 2, and 4 h) following CLSM analysis. Based on the imaging results (Figure 3A), it was observed that the SS-ODN-FAM probe was rapidly dispersed in the cytoplasm within 0.5 h. The probe concentration gradually increased as the fluorescence intensity in the cells increased with the prolonged incubation time. At first, when the intracellular probe concentration was low, the fluorescence signal basically existed in the form of point-like stars. Subsequently, the punctate stars amplified and localized in the form of plates. The complex formed by Lipo2000 and ODN-FAM adhered to the culture dish and cell surface, which could not be effectively cleaned up at the post-treatment stage, resulting in a large amount of extracellular fluorescence interference. Therefore, Lipo2000-mediated cellular uptake experiments were not reported here. Further, the uptake process of different concentrations of SS-ODN-FAM probes was investigated in HeLa S3 cells. Different concentrations of SS-ODN-FAM (0.1–1.0 μM, in DMEM) probe were incubated with HeLa S3 cells at 37 °C for 1 h and analyzed by CLSM. Clearly, there was a significant concentration-dependent cellular uptake of the SS-ODN-FAM probe (Figure 4). At a low concentration (0.1 μM), only weak trace fluorescence excitation was found in the cells, and at a high concentration (1.0 μM), a strong fluorescence signal was detected in the cells. Fluorescence intensity analysis (Figure 4B) also confirmed the strong linear correlation between intracellular fluorescence intensity and the concentration of the probe. The above results fully confirmed the excellent potential of the disulfide unit-masked system for oligonucleotide drug delivery. Compared with commercial transfection reagents with serious challenges in the formation of homogeneous complexes, this system does not require the formation of additional liposome-ODN complexes, and the construction of disulfide unit-masked couplings perfectly avoids this defect.

#### 2.2.2. Cellular Cytotoxicity Evaluation of Disulfide Unit Conjugation Compared with Lipo2000 Reagent

**Figure 5:**
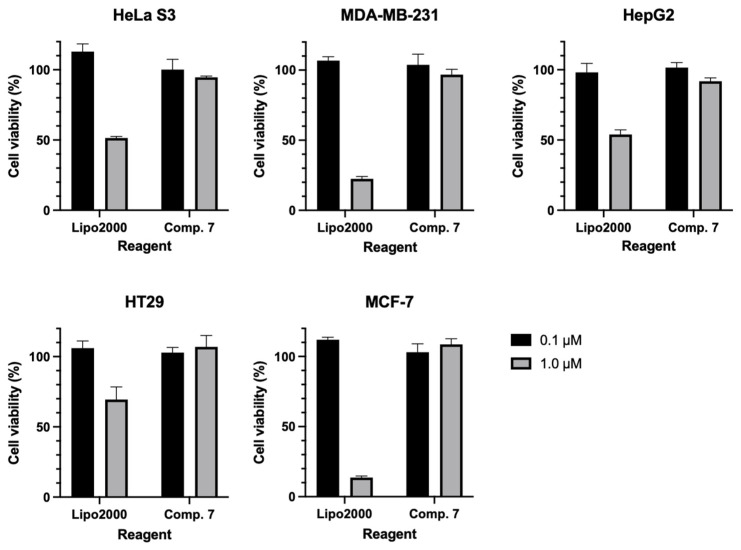
The cytotoxicity of disulfide unit system for intermediate **7** and Lipo2000 using CCK-8 assay.

To evaluate the cellular toxicity between the disulfide unit system and Lipo2000, the CCK-8 assay was implemented. To avoid the effect of fluorophores, synthetic intermediate **7** was used in this experiment. Specifically, two concentrations of 0.1 and 1.0 µM were used for the disulfide unit system. Lipo2000 used two equivalents of the corresponding ODN, at which point a stable complex could be formed in basic cellular experiments. Clearly, both the disulfide unit compound backbone and its intracellular metabolites exhibit friendly cytotoxicity to various tumor cells, even at high concentrations (Figure 5). In contrast, Lipo2000 is relatively safe at low concentrations (0.1 µM) and exhibits significant cytotoxicity as the concentration increases (1.0 µM). The above results fully demonstrate the safety of this disulfide unit-masked delivery vehicle.

#### 2.2.3. Cellular Uptake of the SS-ODN-FAM Probe under Thiol Reagent Treatment

**Figure 6:**
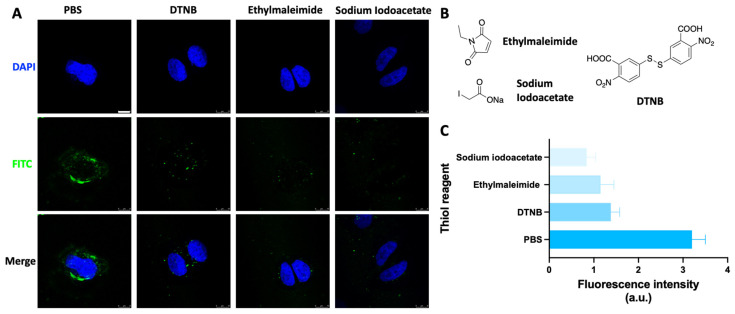
(**A**) CLSM images of HeLa S3 cells that were preincubated with thiol reagent (1.2 mM, 0.5 h), then incubated with an SS-ODN-FAM probe (1 μM, 1 h). The nuclei were stained with DAPI. (**B**) The detailed structure of different thiol reagents, such as ethylmaleimide, sodium iodoacetate, and DTNB. (**C**) Quantitative analysis of fluorescence intensity. Scar bar: 8 µm. The blue marks represent DAPI staining. The green marks represent the fluorescent signal of FAM.

To further explore the delivery mechanism of disulfide units, the HeLa S3 cells were treated with different thiol reagents before incubation with the SS-ODN-FAM probe (Figure 6). As mentioned in the introduction, the first and most important aspect of disulfide unit delivery is the reaction and covalent binding of the disulfide unit in the oligonucleotide drug to thiol groups on the cell membrane. Obviously, the number and redox state of thiol groups on the cell membrane determines the effectiveness of this system for large oligonucleotide drug delivery. Therefore, several chemical reagents that can alter the state of thiol groups on the cell membrane were used to analyze the effect on cellular uptake by CLSM at the cellular level. N-Ethylmaleimide [27] (Figure 6B) and sodium iodoacetate [28] (Figure 6B), the alkylating reagents that react with thiol groups, form stable covalent sulfide ether bonds with the thiol groups allowing them to be permanently closed to prevent disulfide bond formation. Incubation of N-ethylmaleimide and sodium iodoacetate with HeLa S3 cells for half an hour ahead of time reduced SS-ODN-FAM probe cellular uptake by 64% and 74% (Figure 6A,C), respectively. The 5,5′-dithiobis-(2-nitrobenzoic acid) (DTNB, Figure 6B) [29] can be oxidized to mixed disulfides by reacting with free thiol groups. Having incubated DTNB with HeLa S3 cells half an hour earlier, SS-ODN-FAM probe cell uptake capacity was reduced by 57% (Figure 6A,C). The above results fully demonstrate that the SS-ODN-FAM probe indeed delivers oligonucleotide drugs into the cell by dynamic covalent thiol exchange with thiol groups on the cell membrane, and not in an endocytosis manner.

#### 2.2.4. Distribution of SS-ODN-FAM Probe In Vivo

**Figure 7:**
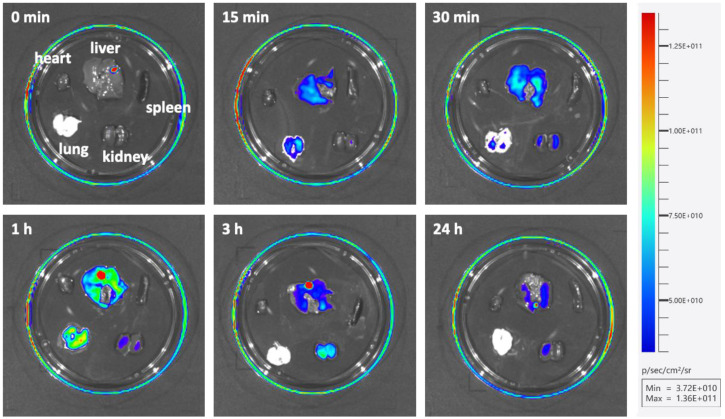
Distribution of SS-ODN-FAM probe in vivo.

Finally, in order to observe the distribution of the SS-ODN-FAM probe in vivo, animal experiments were performed using mice. The SS-ODN-FAM probe (200 μL, 5 mg/kg) was injected into male TCR mice (six weeks old) by tail vein injection, and uninjected mice were used as controls. Mice were sacrificed at the indicated time points after administration (15 min, 30 min, 1 h, 3 h, and 24 h) and all major organs (heart, liver, spleen, lung, and kidney) were collected for fluorescence imaging using the Tanon ABL-X5 Pro Small Animal Live Imaging System. It was found that the probe was rapidly enriched in the liver and lung organs (15 min, Figure 7) after injection, peaking at around 1 h. It was then metabolized and excreted via the kidneys. The probe in the lungs was first metabolized rapidly, leaving essentially no residue after 3 h, when renal work intensified to complete probe excretion. The metabolism of the probe in the liver was relatively slower, and some traces of the probe could still be found in the liver 24 h after injection. Correspondingly, this was accompanied by little metabolism in the kidneys. The distribution of the probe was largely absent in the heart and spleen. These results suggest that the disulfide unit-masked system has the potential to deliver oligonucleotide drugs that can silence genes associated with lung and liver diseases well, especially in the liver site.

## 3. Materials and Methods

### 3.1. General Method

All chemicals were purchased from commercial suppliers. The ^1^H and ^13^C NMR spectra of synthesized compounds were characterized on a 400 MHz spectrometer (Bruker, Billerica, MA, USA), and high-resolution mass spectra (HRMS) were recorded on a mass spectrometer (Waters, Milford, MA, USA). The fluorescence images of cultured cancer cells were captured by a confocal laser scanning microscope (CLSM, Leica, Wetzlar, Germany, TCS SP8) and analyzed by ImageJ (https://imagej.net/software/imagej/ accessed on 22 July 2024). The CCK-8 assay was performed on a multifunctional microplate reader (Synergy H1, Bio-Tek, Bio-Tek, Winooski, VT, USA). The distribution of the SS-ODN-FAM probe in vivo was analyzed by the Tanon ABL-X6 Small Animal Live Imaging System (Tanon, Shanghai, China).

### 3.2. General Method for Preparation of Disulfide Unit Phosphoramidite Monomer

*Synthesis of 3-(((2-(tert-butyldisulfaneyl)ethoxy)carbonyl)amino)propyl 4-methylbenzenesulfonate (* **2** *)*

To a dried flask was added compound **1** (3.70 g, 13.82 mmol) in dry dichloromethane (50 mL) under a N_2_ atmosphere. Dried pyridine (2.79 mL, 34.55 mmol) and 4-toluenesulfonyl chloride (6.60 g, 34.55 mmol) were successively added to the solvent under an ice bath. The reaction mixture was stirred at room temperature overnight. After the addition of water (20 mL) and a saturated aqueous NH_4_Cl solution (10 mL), the mixture was extracted with CH_2_Cl_2_ (3 × 50 mL). The combined organic extracts were dried over Na_2_SO_4_ and concentrated in vacuo before the purification of the crude product by column chromatography (petroleum ether: ethyl acetate = 60:10), which yielded compound **2** as a colorless oil (5.65 g, 13.41 mmol, 97%).

*Synthesis of Methyl 3,4,5-tris(3-(((2-(tert-butyldisulfaneyl)ethoxy)carbonyl)amino)propoxy)benzoate (* **3** *)*

Compound **2** (5.65 g, 13.41 mmol) was added to a solution of methyl 3,4,5-trihydroxybenzoate (0.62 g, 3.35 mmol) and K_2_CO_3_ (1.85 g, 13.41 mmol) in dry DMF (20 mL). The resulting mixture was stirred at 80 °C overnight under a N_2_ atmosphere. Next, ethyl acetate (200 mL) was added, and the organic phase was washed with brine (3 × 30 mL) and dried over Na_2_SO_4_ before the solvent was removed under reduced pressure. Purification by column chromatography (petroleum ether: ethyl acetate = 20:1) yielded the title compound as a pale-yellow oil (**3**, 2.0 g, 2.15 mmol, 64%). ^1^H NMR (400 MHz, CDCl_3_) δ 7.29 (s, 2H), 4.32–4.29 (m, 8H), 3.91 (s, 3H), 3.49–3.36 (m, 8H), 2.93–2.90 (m, 8H), 2.08–1.98 (m, 6H), 1.34 (s, 27H). ESI-HRMS (*m/z*): Calcd. For C_28_H_66_N_3_O_11_S_6_ [M + H]^+^: 932.3016, Found: 932.3017.

*Synthesis of 3,4,5-tris(3-(((2-(tert-butyldisulfaneyl)ethoxy)carbonyl)amino)propoxy)benzoic acid (* **4** *)*

To a solution of compound **3** (2.0 g, 2.15 mmol) in MeOH/THF (1:1, 22 mL) was added 2N aqueous NaOH (0.43 g, 10.75 mmol). The resulting mixture was stirred at room temperature for 5 h. After adding water (15 mL), the pH was neutralized to 4.0 by 1N HCl solution. The mixture was extracted with EtOAc (3 × 50 mL). The combined organic extracts were dried over Na_2_SO_4_ and concentrated in vacuo to yield compound **4** as a colorless oil (1.84 g, 2.00 mmol, 93%) without purification for the next step.

*Synthesis of methyl 6-(3,4,5-tris(3-(((2-(tert-butyldisulfaneyl)ethoxy)carbonyl)amino)propoxy)benzamido)hexanoate (* **5** *)*

To a dried flask was added compound **4** (0.2 g, 0.22 mmol) and DIPEA (113 μL, 0.65 mmol) in dry DMF (2.2 mL) under a N_2_ atmosphere. HOBt (59 mg, 0.44 mmol) and HATU (100 mg, 0.26 mmol) were successively added to the solvent, followed by the addition of methyl 6-aminohexanoate hydrochloride (80 mg, 0.44 mmol) after 30 min. The reaction mixture was stirred at room temperature overnight. After adding water (10 mL), the mixture was extracted with CH_2_Cl_2_ (3 × 10 mL). The combined organic extracts were dried over Na_2_SO_4_ and concentrated in vacuo before the purification of the crude product by column chromatography (CH_2_Cl_2_: MeOH = 70:1), which yielded compound **5** as a colorless oil (0.17 g, 0.16 mmol, 74%). ^1^H NMR (400 MHz, CDCl_3_) δ 7.05 (s, 2H), 4.33–4.29 (m, 6H), 4.16–4.10 (m, 6H), 3.68 (s, 3H), 3.49–3.40 (m, 8H), 2.94–2.91 (m, 6H), 2.36 (t, *J* = 7.3 Hz, 2H), 2.09–1.97 (m, 6H), 1.70–1.65 (m, 6H), 1.35 (s, 27H). ESI-HRMS (*m/z*): Calcd. For C_44_H_77_N_4_O_10_S_6_ [M + H]^+^: 1045.3857, Found: 1045.3856.

*Synthesis of 6-(3,4,5-tris(3-(((2-(tert-butyldisulfaneyl)ethoxy)carbonyl)amino)propoxy)benzamido)hexanoic acid (* **6** *)*

To a solution of compound **5** (0.44 g, 0.42 mmol) in MeOH/THF (1:1, 4 mL) was added 2N aqueous NaOH (84 mg, 2.10 mmol). The resulting mixture was stirred at room temperature for 5 h. After adding water (15 mL), the pH was neutralized to 4.0 by 1N HCl solution. The mixture was extracted with EtOAc (3 × 30 mL). The combined organic extracts were dried over Na_2_SO_4_ and concentrated in vacuo before the purification of the crude product by column chromatography (CH_2_Cl_2_: MeOH = 50:1), which yielded compound **6** as a colorless oil (248 mg, 0.24 mmol, 57%). ^1^H NMR (400 MHz, CDCl_3_) δ 7.09 (s, 2H), 4.28–4.19 (m, 6H), 4.07–4.00 (m, 6H), 3.42–3.30 (m, 8H), 2.85–2.82 (m, 6H), 2.35–2.30 (m, 2H), 2.01–1.86 (m, 6H), 1.61–1.55 (m, 6H), 1.26 (s, 27H). ^13^C NMR (101 MHz, CDCl_3_) δ 177.83, 176.68, 167.18, 158.12, 156.62, 156.42, 152.28, 139.91, 130.42, 130.21, 106.27, 105.74, 104.94, 77.27, 71.88, 67.28, 67.09, 66.80, 63.95, 63.19, 62.89, 48.00, 39.25, 39.04, 38.60, 33.29, 30.00, 29.88, 29.87, 29.73, 29.56, 29.41, 29.29, 28.22, 25.87, 23.75, 23.38, 20.66. ESI-HRMS (*m/z*), calcd. C_43_H_75_N_4_O_12_S_6_ [M + H]^+^: 1031.3700, Found: 1031.3692.

*Synthesis of bis(2-(tert-butyldisulfaneyl)ethyl) (((2-(3-(((2-(tert-butyldisulfaneyl)ethoxy)carbonyl)amino)propoxy)-5-((6-((3-hydroxypropyl)amino)-6-oxohexyl)carbamoyl)-1,3-phenylene)bis(oxy))bis(propane-3,1-diyl))dicarbamate (* **7** *)*

To a dried flask was added compound **6** (200 mg, 0.19 mmol) and DIPEA (66 μL, 0.76 mmol) in dry DMF (1.9 mL) under a N_2_ atmosphere. HOBt (51 mg, 0.38 mmol) and HATU (144 mg, 0.38 mmol) were successively added to the solvent, followed by the addition of 3-amino-1-propanol (29 mg, 0.38 mmol) after 30 min. The reaction mixture was stirred at room temperature overnight. After adding water (3 mL), the mixture was extracted with CH_2_Cl_2_ (3 × 10 mL). The combined organic extracts were dried over Na_2_SO_4_ and concentrated in vacuo before the purification of the crude product by column chromatography (CH_2_Cl_2_: MeOH = 15:1), which yielded compound **7** as a colorless oil (180 mg, 0.16 mmol, 84%). ^1^H NMR (400 MHz, CDCl_3_) δ 7.00 (s, 2H), 4.24–4.19 (m, 6H), 4.06–4.00 (m, 6H), 3.53 (t, *J* = 6.0 Hz, 2H), 3.38–3.30 (m, 10H), 2.85–2.81 (m, 6H), 2.16 (t, *J* = 6.0 Hz, 2H), 1.99–1.88 (m, 6H), 1.64–1.55 (m, 6H), 1.35–1.33 (m, 2H), 1.26 (s, 27H). ^13^C NMR (101 MHz, CDCl_3_) δ 174.26, 167.23, 156.48, 156.43, 152.32, 140.08, 130.15, 105.84, 77.27, 71.79, 67.20, 63.03, 62.89, 59.57, 48.01, 47.99, 39.72, 39.26, 39.13, 38.89, 38.51, 36.57, 36.21, 32.07, 29.88, 29.49, 28.94, 26.10, 24.85. ESI-HRMS (*m/z*): Calcd. For C_46_H_82_N_5_O_12_S_6_ [M + H]^+^: 1088.4279 Found: 1088.4270.

*Synthesis of bis(2-(tert-butyldisulfaneyl)ethyl) (((2-(3-(((2-(tert-butyldisulfaneyl)ethoxy)carbonyl)amino)propoxy)-5-((6-((3-(((2-cyanoethoxy)(diisopropylamino)phosphaneyl)oxy)propyl)amino)-6-oxohexyl)carbamoyl)-1,3-phenylene)bis(oxy))bis(propane-3,1-diyl))dicarbamate (* **8** *)*

Under a N_2_ atmosphere, to a solution of compound **7** (180 mg, 0.17 mmol) in CH_2_Cl_2_ (3.4 mL) was added *N,N*-diisopropylethylamine (173 μL, 0.99 mmol) and 2-cyanoethyl-*N*,*N*-diisopropylchlorophosphoramidite (110 μL, 0.50 mmol) and stirred at 0 °C for 1 h. The reaction mixture was diluted with EtOAc (20 mL) and washed with saturated aqueous NaHCO_3_ (20 mL) and brine (20 mL). The organic layer was dried over Na_2_SO_4_, concentrated under vacuum, and purified by column chromatography (CH_2_Cl_2_/MeOH = 100:2) to give a pale-yellow oil **8** (150 mg, 68%). ^31^P NMR (162 MHz, CDCl_3_) *δ* 147.77.

### 3.3. Preparation of Disulfide Unit-Conjugated Oligonucleotide (SS-ODN-FAM)

The 19-mer ODN 5′-SS-AATGGGGAGAAGGAGAAGG-FAM-3′ (SS = disulfide unit, SS-ODN-FAM) was synthesized on an automated DNA synthesizer (Nihon Techno Service Co., Ltd.) using standard phosphoramidite chemistry. Cleavage from Controlled Pore Glass (CPG) was achieved by treatment with 28% ammonia aqueous solution at 55 °C overnight, followed by reverse-phase high-performance liquid chromatography (HPLC) purification [column, Waters XBridge C18, 4.6 × 150 mm; solvents, A: 0.1 M triethylamine acetate buffer (pH 7.0), B: CH_3_CN, linear gradient: B for 0–30%/25 min, flow rate: 1.0 mL/min, UV: 260 nm, column oven: 60 °C].

### 3.4. General Protocol for Cell Culture

The various cancer cells (HeLa S3, MDA-MB-231, HepG2, MCF-7, and HT29, kindly provided by Wuhan Pricella Biotechnology Co., Ltd., Wuhan, China) were grown in a complete medium (DMEM containing 10% FBS, 100 units/mL of penicillin, and 100 μg/mL of streptomycin, Adamas life, Shanghai, China). The cells were cultured in a 5% CO_2_ humidified incubator at 37 °C. The cells were harvested by being treated with 0.1% trypsin (Gibco, Waltham, MA, USA, diluted with PBS) and passaged once in 3–4 days.

### 3.5. Time-Dependent Fluorescence Microscopy Measurement of the Cancer Cells Treated with the SS-ODN-FAM Probe and Lipo2000/ODN-FAM Complex

The various cancer cells (HeLa S3, MDA-MB-231, HepG2, HT29, and MCF-7, 1 × 10^4^ cells) were seeded onto 35 mm glass-bottom culture dishes (Biosharp, Guangzhou, China) and grown in a complete medium (DMEM, high glucose, 10% FBS, 1% antibiotics) 24 h before probe treatment. The cells were treated with SS-ODN-FAM (1 µM) probe in DMEM at 37 °C for 0.5, 1.0, 2.0, and 4.0 h. As the control, transfection reagent Lipo2000 (Biosharp) was used to form the Lipo2000/ODN-FAM complex with ODN-FAM according to the manual (L:N = 2:1, 1 µM). After that, the cells were washed with PBS (1 mL × 3) and fixed with 4% PFA for 15 min. All cells were stained with DAPI for 15 min followed by PBS wash (1 mL × 3). The cells were immediately analyzed using a fluorescence microscope (Leica TCS SP8).

### 3.6. Concentration-Dependent Fluorescence Microscopy Measurement of the Cancer Cells Treated with the SS-ODN-FAM Probe

The HeLa S3 cells (1 × 10^4^ cells) were seeded onto 35 mm glass-bottom culture dishes (Biosharp) and grown in a complete medium (DMEM, high glucose, 10% FBS, 1% antibiotics) 24 h before probe treatment. The cells were treated with SS-ODN-FAM (1.0, 0.4, and 0.1 µM) probe in DMEM at 37 °C for 1.0 h. After that, the cells were washed with PBS (1 mL × 3) and fixed with 4% PFA for 15 min. All the cells were stained with DAPI for 15 min followed by PBS wash (1 mL × 3). The cells were immediately analyzed using a fluorescence microscope (Leica TCS SP8).

### 3.7. CCK-8 Assay

The various cancer cells (HeLa S3, MDA-MB-231, HepG2, HT29, and MCF-7, 3000–5000 cells/well) were seeded onto a 96-well plate (Labselect, Hefei, China) and grown in a complete medium (DMEM, high glucose, 10% FBS, 1% antibiotics, 100 µL) 24 h before probe treatment. The cells were treated with **7** (0.1 and 1.0 µM, 100 µL) and Lipo2000 (2 equivalent of corresponding ODN, 100 µL), respectively, in DMEM at 37 °C for 72 h. The medium was removed, and 20 μL of CCK-8 solution (Adamas life, Shanghai, China) was added to each plate; the cells were then incubated at 37 °C for 4 h. A multifunctional microplate reader (Synergy H1, Bio-Tek, Winooski, VT, USA) was used to record the absorbance of the solution in each well at 450 nm.

### 3.8. Fluorescence Microscopy Measurement of the Cancer Cells Pretreated with Thiols Reagents

The HeLa S3 cells (1 × 10^4^ cells) were seeded onto 35 mm glass-bottom culture dishes (Biosharp) and grown in a complete medium (DMEM, high glucose, 10% FBS, 1% antibiotics) 24 h before probe treatment. The cells were pretreated with N-Ethylmaleimide (1.2 mM), sodium iodoacetate (1.2 mM), and DTNB (1.2 mM), respectively, in DMEM at 37 °C for 0.5 h. After that, the cells were then treated with SS-ODN-FAM (1 µM) probes in DMEM at 37 °C for 1 h. The cells were washed with PBS (1 mL × 3) and fixed with 4% PFA for 15 min. All the cells were stained with DAPI for 15 min followed by PBS wash (1 mL × 3). The cells were immediately analyzed using a fluorescence microscope (Leica TCS SP8).

### 3.9. In Vivo Distribution of SS-ODN-FAM Probe

All animal experimental protocols were approved by the Animal Research and Care Committee of Nantong University. Male ICR mice (6 weeks old) were purchased from the Model Animal Research Center affiliated with the Experimental Animal Center of Nantong University (Nantong, China). The in vivo biodistribution of SS-ODN-FAM in mice was investigated by the Tanon ABL-X5 Pro series small animal live imaging system (Shanghai, China). The mice were randomly grouped and intravenously injected with 200 µL of SS-ODN-FAM at an identical dose of 5 mg/kg. The uninjected group was a control. Mice were sacrificed after the specified time points at 15 min, 30 min, 1 h, 3 h, and 24 h. All the major organs (heart, liver, spleen, lungs, and kidneys) were collected for fluorescence measurements after the step of in vivo imaging.

## 4. Conclusions

In conclusion, an oligonucleotide drug delivery system masked by a trivalent disulfide unit with 3,4,5-trihydroxybenzoic acid as its backbone was constructed; in particular, a ring-opening relaxation-type disulfide unit with a lower reactivity of *tert*-butyl substitution was employed. At the cellular level, this system exhibits superior cellular delivery over the commercial nucleic acid transfection reagent Lipo2000 in an endocytosis-independent cellular delivery mechanism. The disulfide unit is coupled directly to one end of the oligonucleotide drug. The system constructed by this method is uniform in size, free of excessive charge, and essentially non-cytotoxic. In particular, this system does not require the process of preparing material–nucleic acid complexes, and the formation of homogeneous complexes is a highly challenging task. Cellular imaging results show that this system can enrich oligonucleotides in the cytoplasm, possessing the ability to deliver oligonucleotide drugs targeting the cytoplasmic genome. In vivo distribution results in mice showed that oligonucleotide can be enriched in liver tissue and may exhibit good delivery of oligonucleotide drugs targeting the liver. Thus, this disulfide unit-masked delivery system has the potential to be an effective delivery platform for large oligonucleotide drugs, and efforts to study its specific therapeutic effects are in progress.

## Data Availability

The raw data supporting the conclusions of this article will be made available by the authors on request.

## Supplementary Materials

The following supporting information can be downloaded at: https://www.mdpi.com/article/10.3390/molecules29174223/s1. Figures S1–S5: NMR and MS data of all compounds; Figure S6: The HPLC analysis of SS-ODN-FAM; Figure S7: The MS analysis of SS-ODN-FAM; Figure S8: CLSM images of MDA-MB-231 cells and HepG2 cells after incubation with 1 μM SS-ODN-FAM probe and Lipo2000/ODN-FAM (1 μM) complex; Figure S9: CLSM images of HT29 cells and MCF-7 cells after incubation with 1 μM SS-ODN-FAM probe and Lipo2000/ODN-FAM (1 μM) complex.
