# Supplementary material for: Trivalent Disulfide Unit-Masked System Efficiently Delivers Large Oligonucleotide
Source: Molecules. 2024 Sep 5;29(17):4223. doi: 10.3390/molecules29174223 (PMC11397113; doi:10.3390/molecules29174223)
Supplement: Supplementary file 1 [file molecules-29-04223-s001.zip › molecules-3142898-supplementary.pdf]

Trivalent Disulfide Unit-Masked System Efficiently Delivers Large Oligonucleotide

Lei Wang *, Xiao Liu, Yiliang Wu, Zhaoyan Ye, Yiru Wang, Shengshu Gao, Hao Gong and Yong Ling *

School of Pharmacy, Jiangsu Province Key Laboratory for Inflammation and Molecular Drug Target,
Nantong University, Nantong 226001, China; lx199803@126.com (X.L.); 13815218649@163.com (Y.W.);
jackcooper1128@163.com (Z.Y.); yrjw779@126.com (Y.W.); 19551223085@163.com (S.G.);
18915458067@163.com (H.G.)
* Correspondence: wanglei2111@ntu.edu.cn (L.W.); lyyy111@ntu.edu.cn (Y.L.)

Table of contents

Figure S1-S5. NMR and MS data of all compounds 2

Figure S6. The HPLC analysis of SS-ODN-FAM 9

Figure S7. The MS analysis of SS-ODN-FAM 9

Figure S8. CLSM images of MDA-MB-231 cells and HepG2 cells after incubation with 1 μM SS-ODN-FAM probe and Lipo2000/ODN-FAM (1 μM) complex. 10

Figure S9. CLSM images of HT29 cells and MCF-7 cells after incubation with 1 μM SS-ODN-FAM probe and Lipo2000/ODN-FAM (1 μM) complex. 10

Methyl 3,4,5-tris(3-(((2-(*tert*-butyldisulfaneyl)ethoxy)carbonyl)amino)propoxy)benzoate (3)

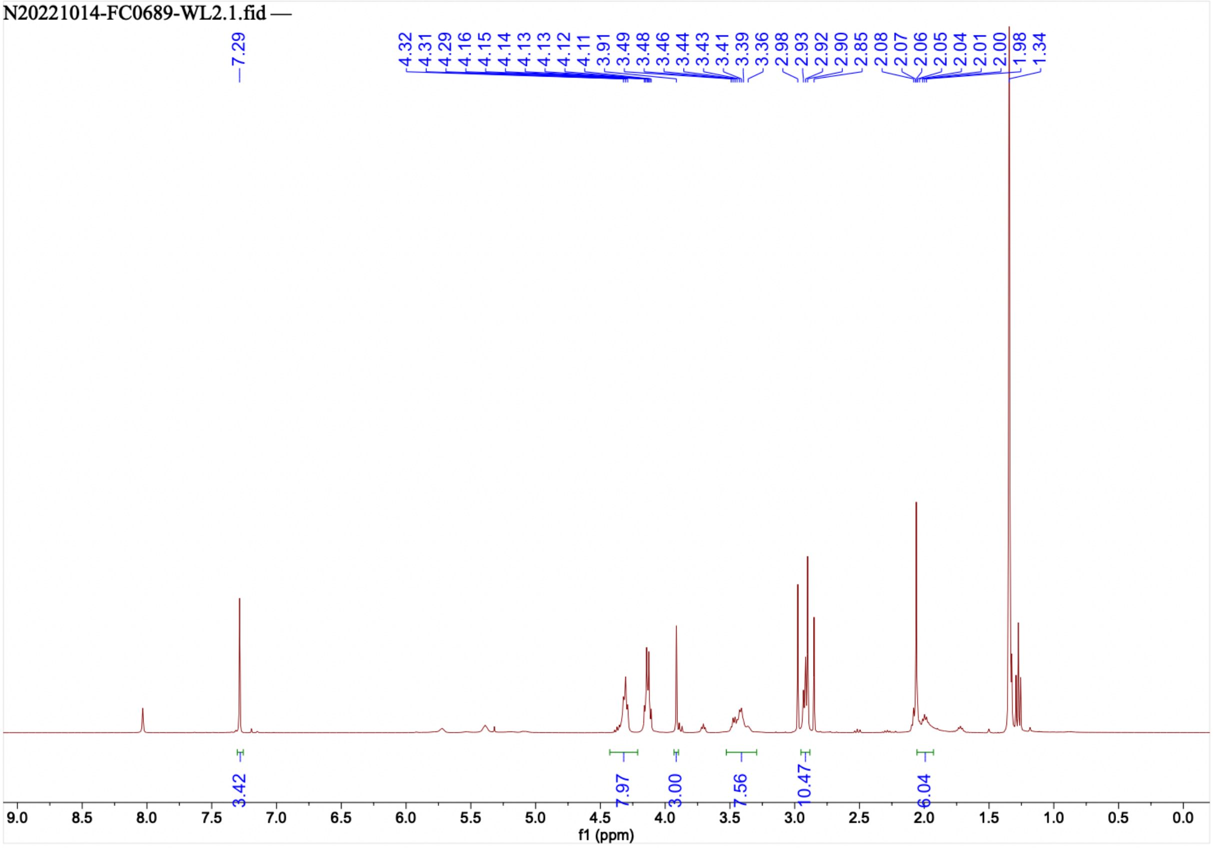

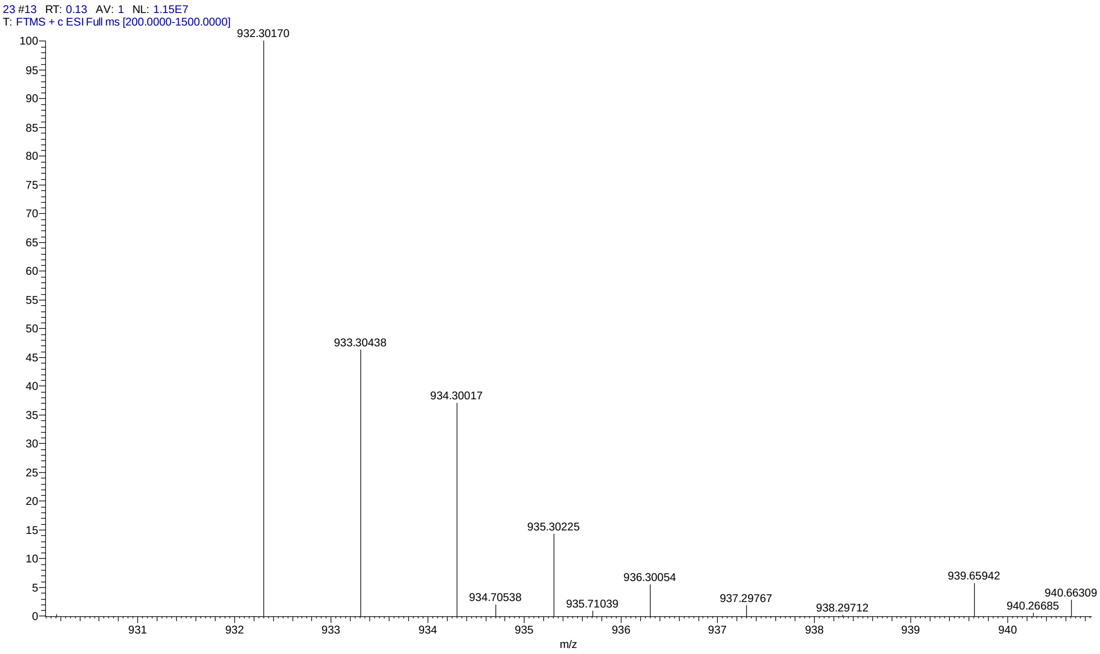

Figure S1. The ^{1}H NMR and HRMS of compound **3**

methyl 6-(3,4,5-tris(3-(((2-(*tert*-butyldisulfaneyl)ethoxy)carbonyl)amino)propoxy)benzamido)hexanoate (5)

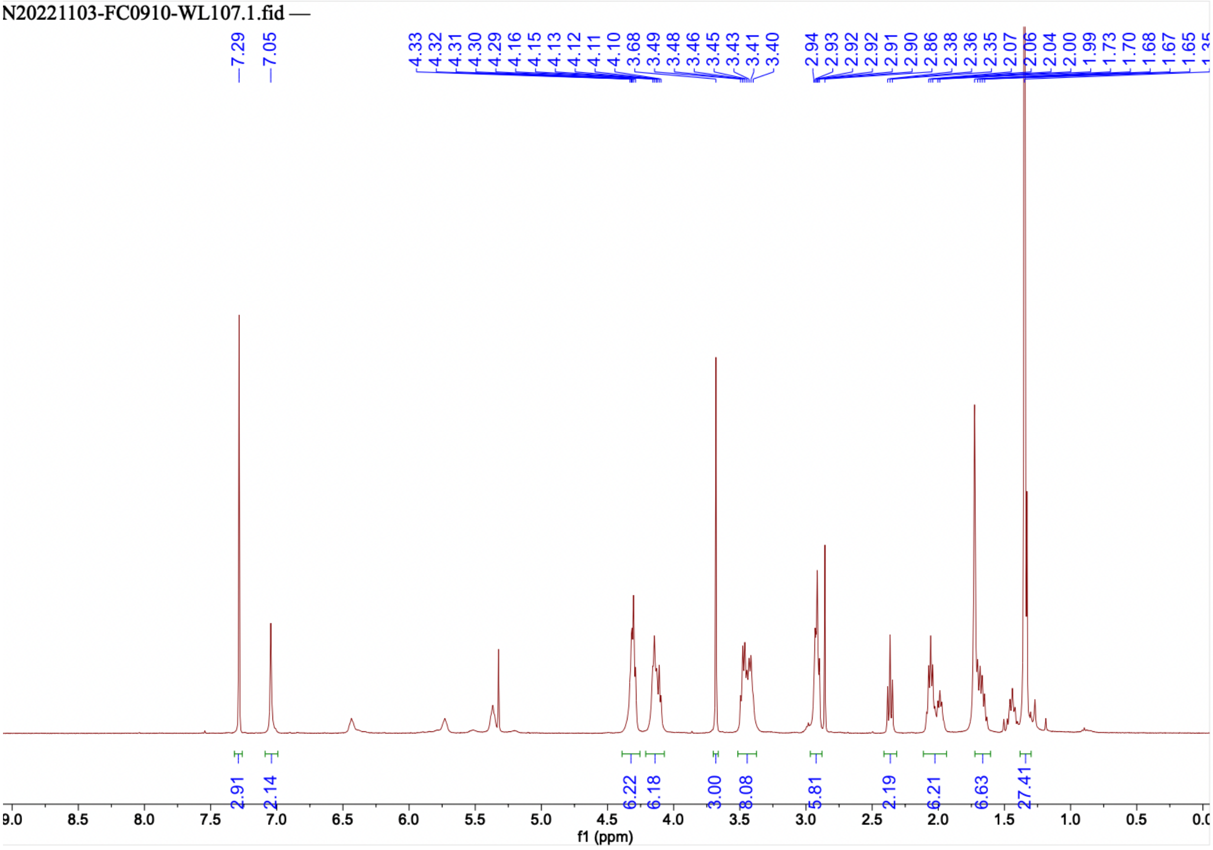

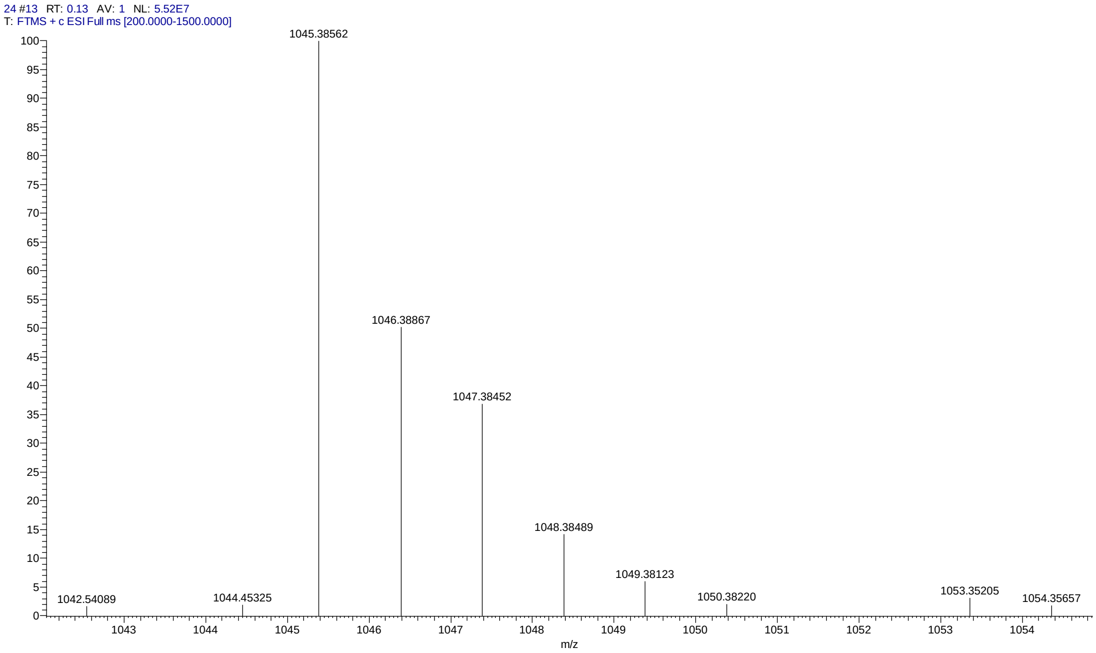

Figure S2. The ^{1}H NMR and HRMS of compound **5**

6-(3,4,5-tris(3-(((2-(*tert*-butyldisulfaneyl)ethoxy)carbonyl)amino)propoxy)benzamido)hexanoic acid (6)

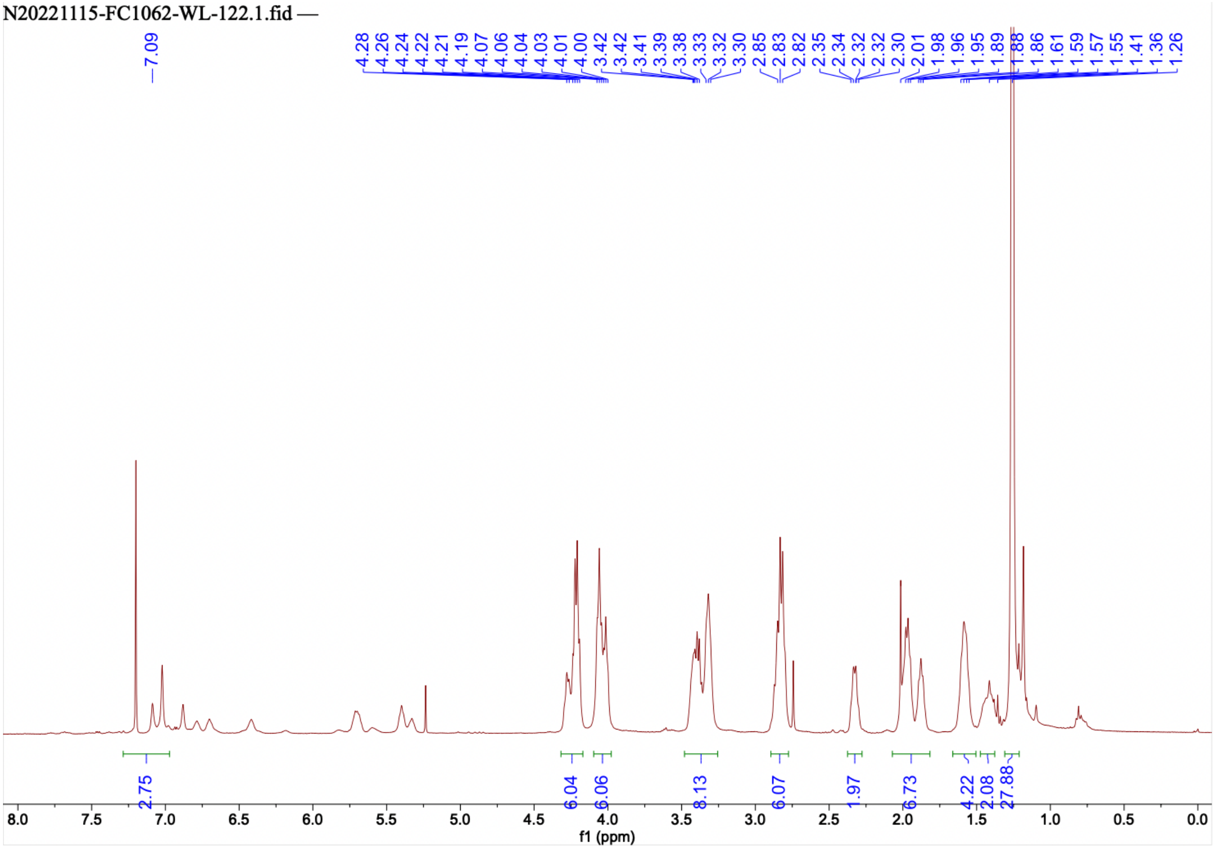

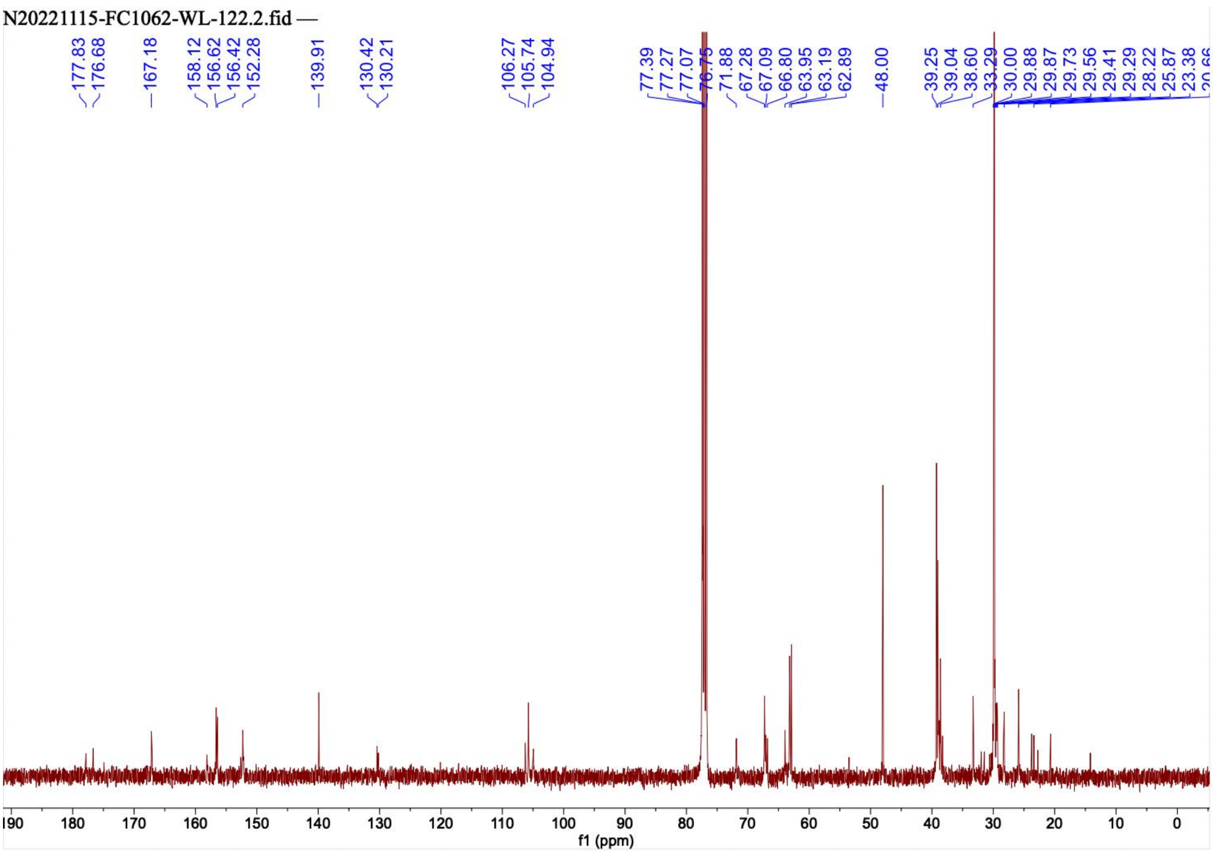

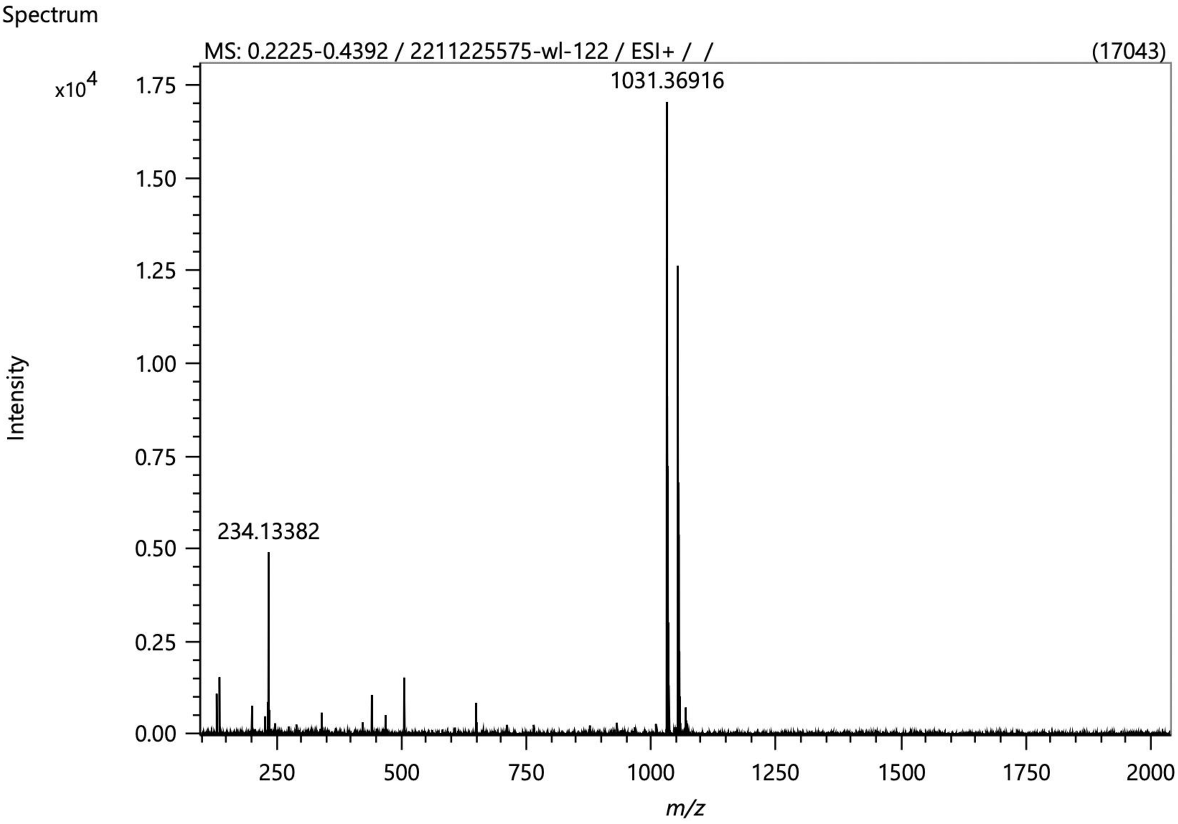

Figure S3. The ^{1}H NMR, ^{13}C NMR and HRMS of compound **6**

bis(2-(*tert*-butyldisulfaneyl)ethyl) (((2-(3-(((2-(*tert*-butyldisulfaneyl)ethoxy)carbonyl)amino)propoxy)-5-((6-((3-hydroxypropyl)amino)-6-oxohexyl)carbamoyl)-1,3-phenylene)bis(oxy))bis(propane-3,1-diyl))dicarbamate (7)

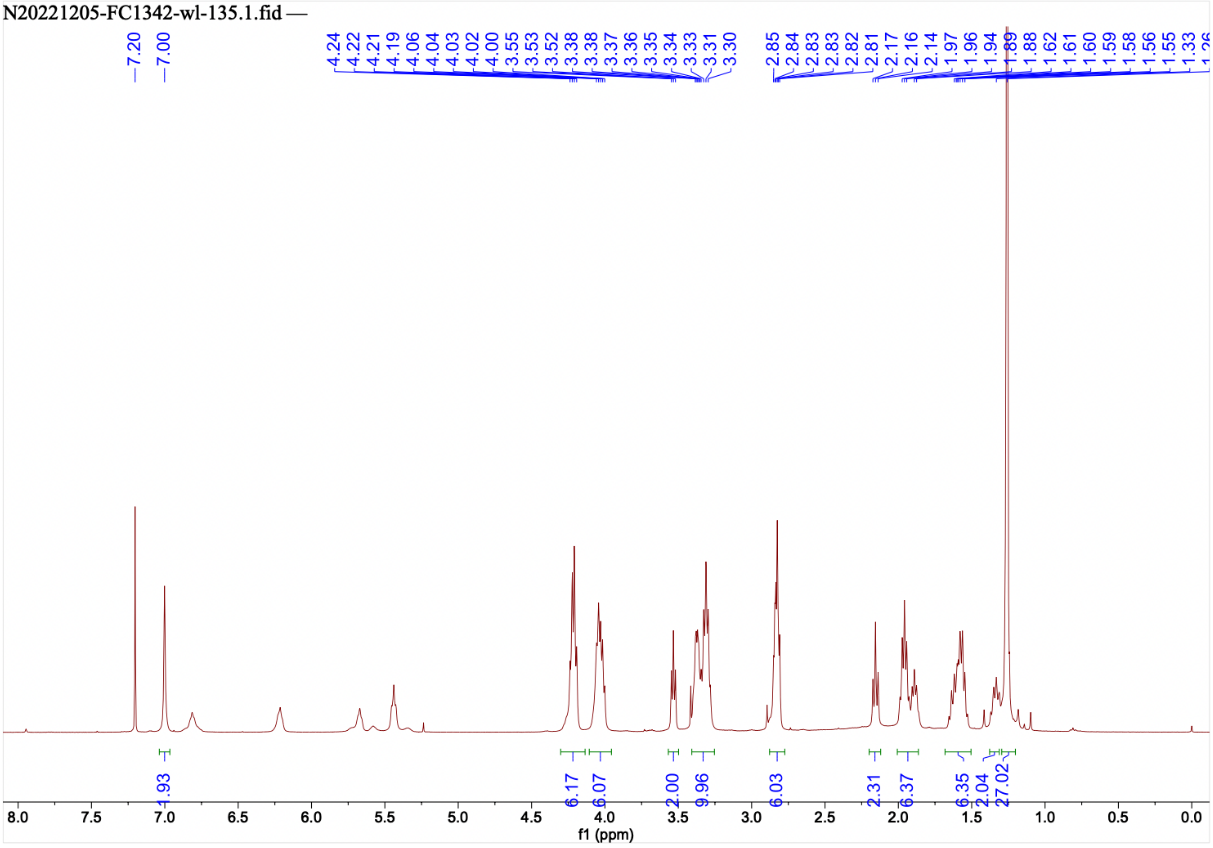

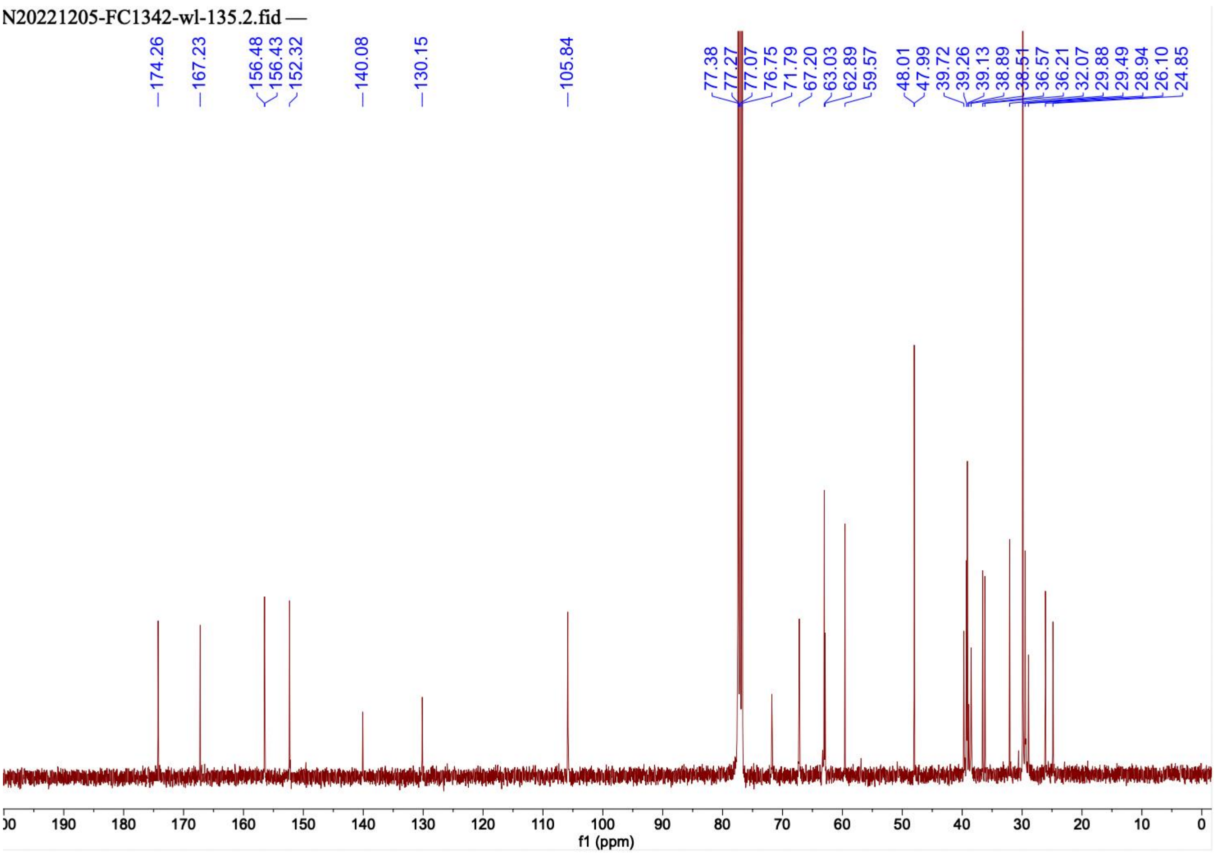

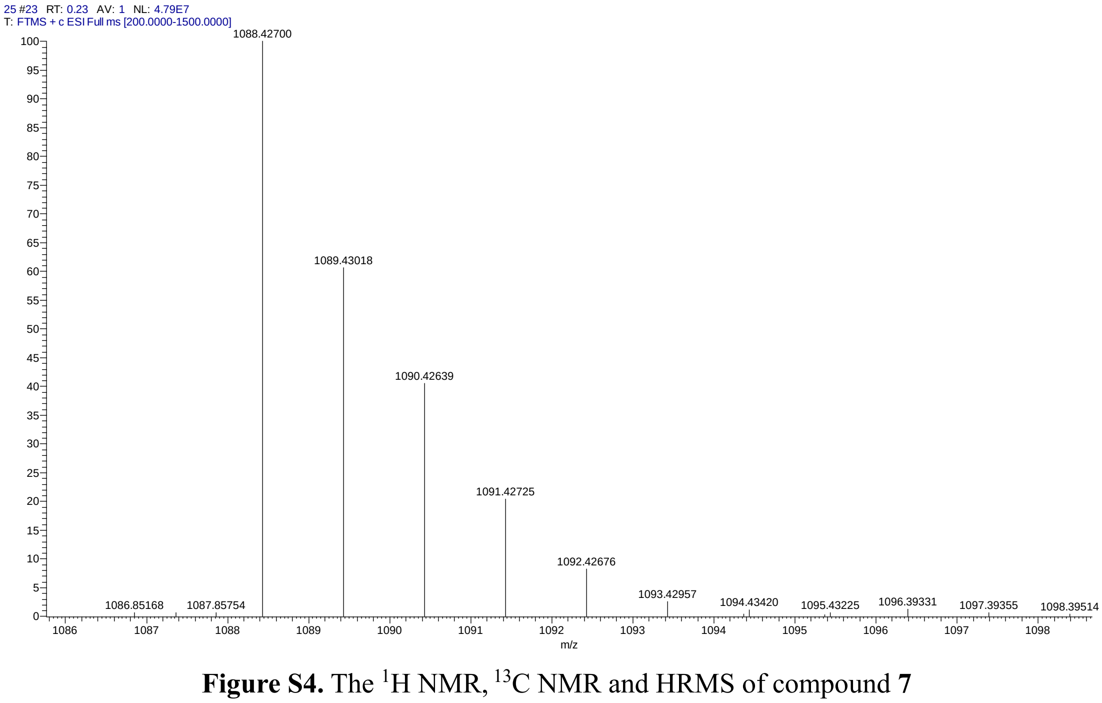

Figure S4. The ^{1}H NMR, ^{13}C NMR and HRMS of compound **7**

bis(2-(tert-butyldisulfaneyl)ethyl) (((2-(3-(((2-(tert-butyldisulfaneyl)ethoxy)carbonyl)amino)propoxy)-5-((6-((3-(((2-cyanoethoxy)(diisopropylamino)phosphaneyl)oxy)propyl)amino)-6-oxohexyl)carbamoyl)-1,3-phenylene)bis(oxy))bis(propane-3,1-diyl))dicarbamate (8)

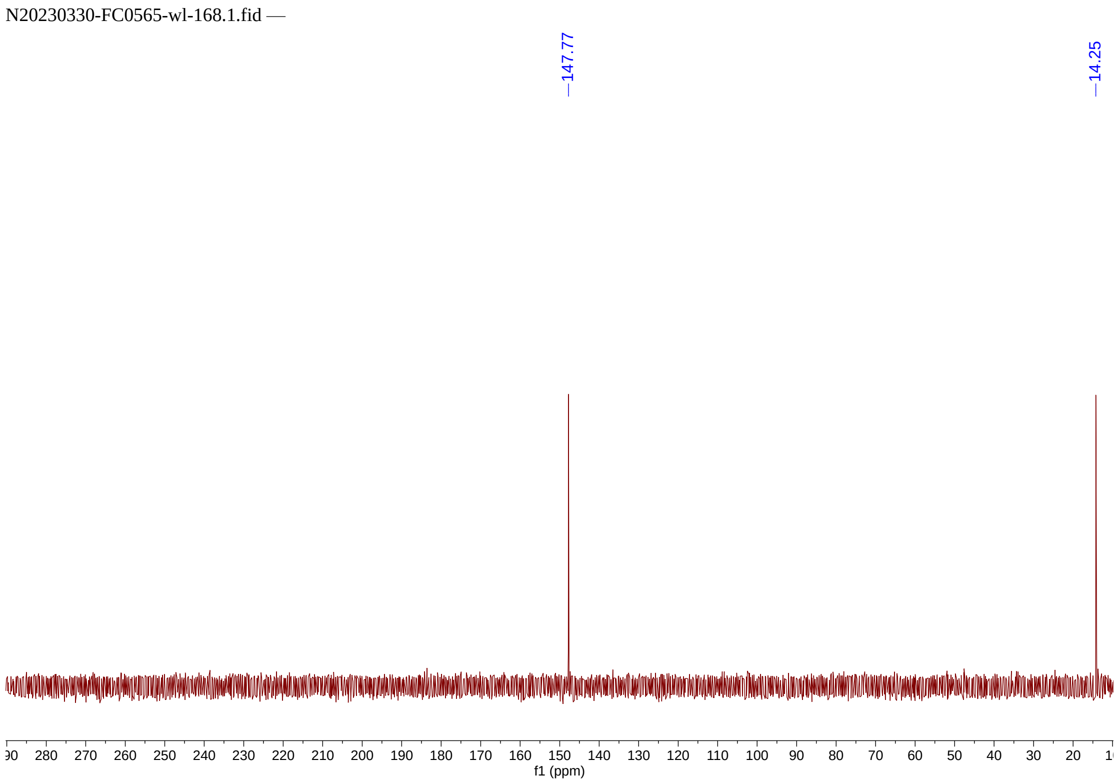

Figure S5. The ^{31}P NMR of compound **8**

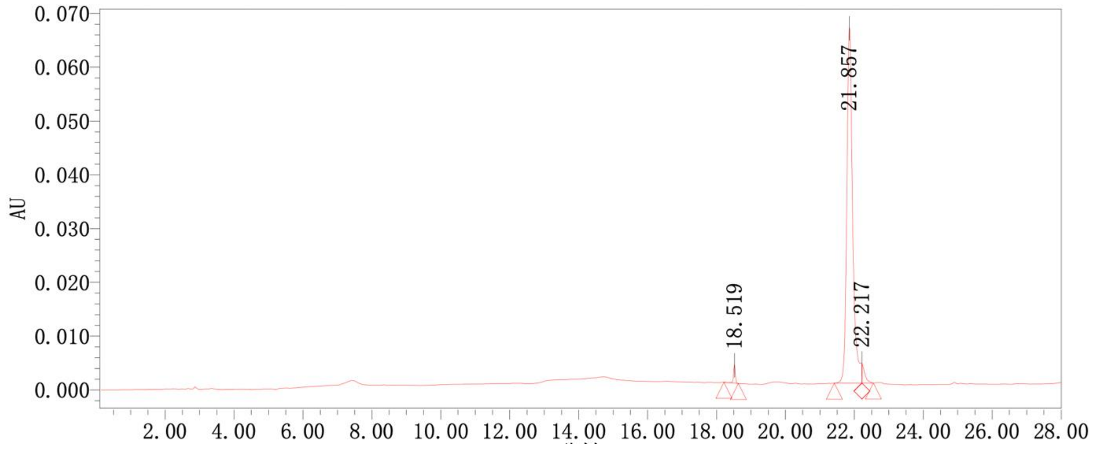

Figure S6. The HPLC analysis of SS-ODN-FAM

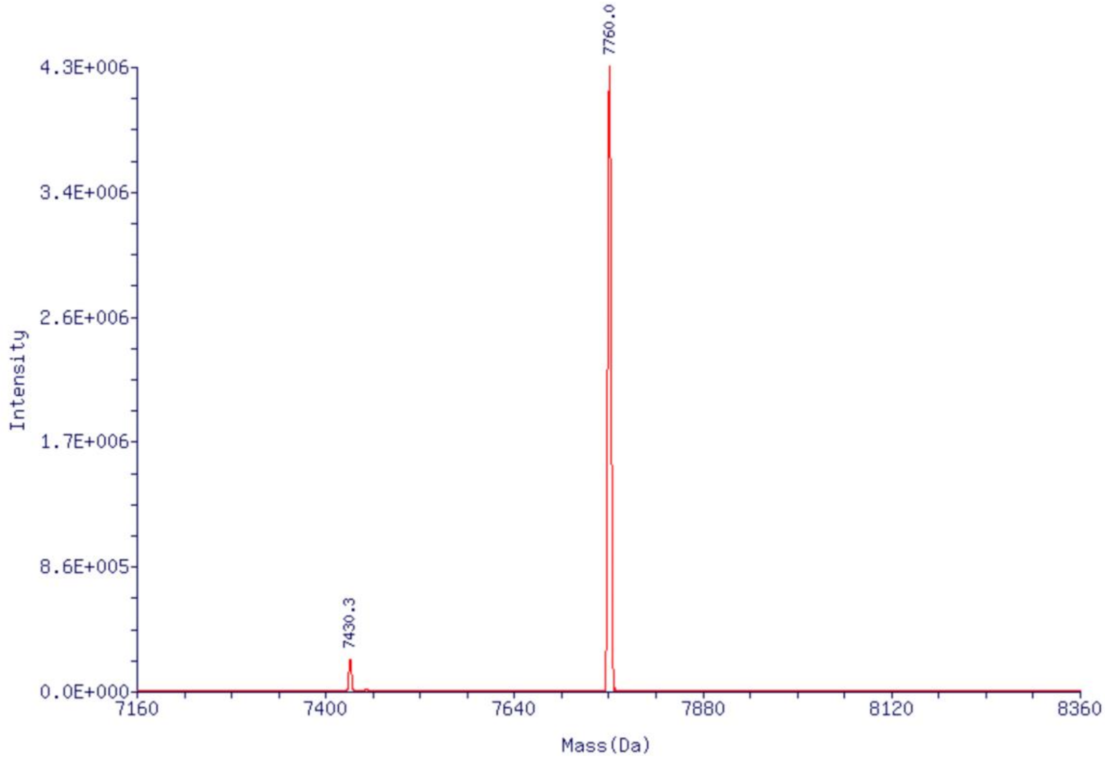

Figure S7. The MS analysis of SS-ODN-FAM

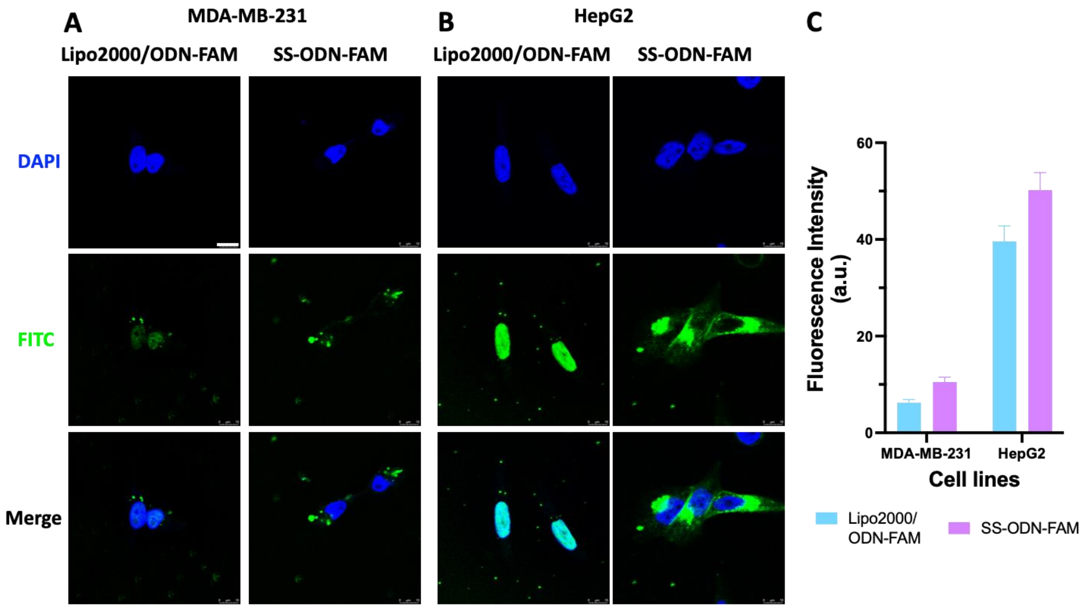

Figure S8. CLSM images of MDA-MB-231 cells (**A**) and HepG2 cells (**B**) after incubation with 1 μM SS-ODN-FAM probe and Lipo2000/ODN-FAM (1 μM) complex for 4 h. The nuclei were stained with DAPI. (**C**) Quantitative analysis of fluorescence intensity. Scar bar: 8 μm.

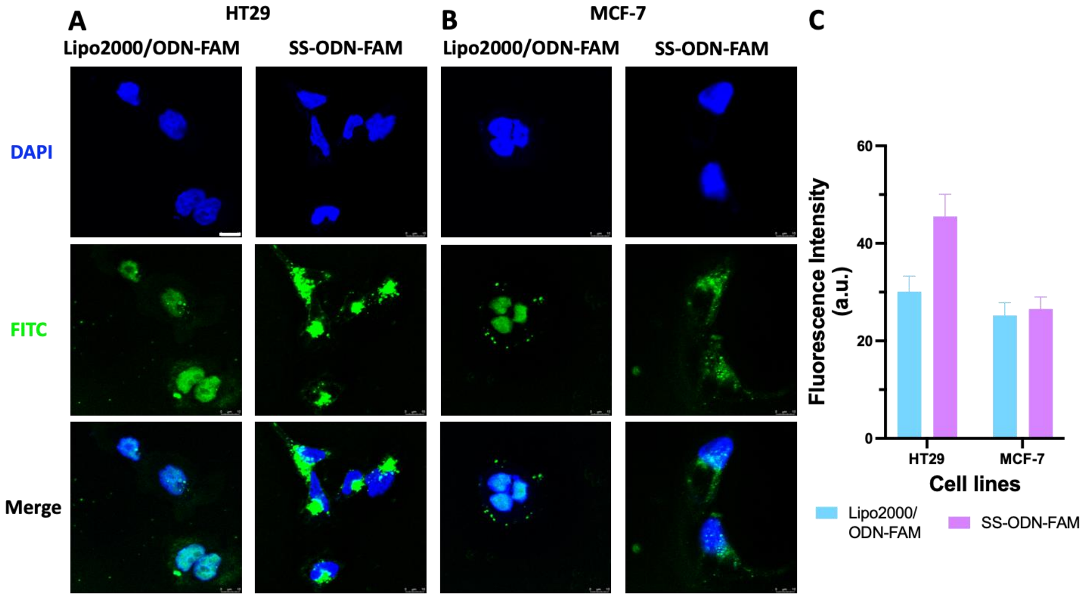

Figure S9. CLSM images of HT29 cells (**A**) and MCF-7 cells (**B**) after incubation with 1 μM SS-ODN-FAM probe and Lipo2000/ODN-FAM (1 μM) complex for 4 h. The nuclei were stained with DAPI. (**C**) Quantitative analysis of fluorescence intensity. Scar bar: 8 μm.
